# Label-Free Bound-States-in-the-Continuum Biosensors

**DOI:** 10.3390/bios12121120

**Published:** 2022-12-02

**Authors:** Man Luo, Yi Zhou, Xuyang Zhao, Yuxiang Li, Zhihe Guo, Xi Yang, Meng Zhang, You Wang, Xiang Wu

**Affiliations:** 1 The Key Laboratory of Micro and Nano Photonic Structures, Department of Optical Science and Engineering, Fudan University, Shanghai 200438, China; 2 The Key Laboratory of Laser Device Technology, China North Industries Group Corporation Limited, Southwest Institute of Technical Physics, Chengdu 640041, China

**Keywords:** bound states in the continuum, label-free, optical biosensors, nanostructures

## Abstract

Bound states in the continuum (BICs) have attracted considerable attentions for biological and chemical sensing due to their infinite quality (*Q*)-factors in theory. Such high-*Q* devices with enhanced light-matter interaction ability are very sensitive to the local refractive index changes, opening a new horizon for advanced biosensing. In this review, we focus on the latest developments of label-free optical biosensors governed by BICs. These BICs biosensors are summarized from the perspective of constituent materials (i.e., dielectric, metal, and hybrid) and structures (i.e., grating, metasurfaces, and photonic crystals). Finally, the current challenges are discussed and an outlook is also presented for BICs inspired biosensors.

## 1. Introduction

Optical biosensors have attracted extensive attention in various research fields [1,2,3]. Generally, there are two distinct types of optical biosensors: labeled and label-free optical biosensors. Compared to labeled optical biosensors, label-free optical biosensors can analyze the biomarkers without changing the properties of analytes, which is a more preferred option. To date, a variety of label-free optical biosensors have been proposed, such as whispering gallery mode sensors, optical fiber sensors, photonics crystal cavities, photonic periodic nanostructures, and so on [4]. These methods pave the way towards high performance biosensing platforms.

Particularly, photonic periodic nanostructures (namely periodic subwavelength resonators, such as grating, metasurfaces, and photonic crystals) can trap the light within the subwavelength regime, and thus substantially enhance the light-matter interaction, promoting the high sensitivity detection of biomarkers. Meanwhile, the periodic nature of these nanostructures can confine the lateral propagation of light, which provides the ability of high-throughput detections with single-molecular resolution [5]. Such abilities enable periodic nanostructures biosensors to provide more promising potential for practical applications than others. Moreover, the thin-film shape of the nanostructures is conducive to lab-on-a-chip platforms, which is necessary for the integration and commercialization of optical biosensors [6,7].

The periodic nanostructures biosensors can be classified into metal-based surface plasmon resonance (SPR) biosensors [8,9,10] and dielectric-based guided-mode resonance (GMR) biosensors [11,12,13,14]. A surface plasmon polariton (SPP) is a surface wave whose electromagnetic field has a strong localization between the metallic film and the medium, and it can overlap well with the surrounding biological solution when used in biosensing. SPR arises when the wave vector of the incident light matches the wave vector of the SPP [10]. SPR biosensors can achieve high bulk sensitivities. However, due to the strong dispersion and absorption of the metal, the quality (*Q*)-factor value and figure of merit (*FOM*) of SPR biosensors are usually limited to a low level (≤10^2^) [15,16,17,18,19]. The *Q* factor associated with the device energy storage stands for the ability to trap the light within the structures, and it is defined as the ratio of the central wavelength to the full width at half maximum (*FWHM*) [20]. The *FOM* represent the overall performances of sensing resolution and sensitivity [21], and these performances are determined by the characteristics of resonance peaks such as the extinction ratio (defined as the ratio of the maximum and the minimum transmission power) [22] and the slope rate (defined as the ratio of the light intensity change to the wavelength shift) [23,24]. Such characteristics act as the indicators of sensing performance.

In dielectric nanostructures, GMR occurs when the guided modes leak out of the structure due to defects on the structure surface and then strongly couple with the external continuous radiation region. Compared to SPR biosensors, GMR biosensors can achieve higher *Q* values based on low-loss dielectric materials. However, it is still difficult for GMR biosensors to achieve high *Q* and high sensitivity simultaneously, because reduction of the GMR linewidth is often accompanied by a more localized electromagnetic field in the structure, which leads to a reduction of the evanescent field distribution in the covering medium, resulting in low electromagnetic energy utilization; thus, the sensitivity of a GMR biosensor will be greatly sacrificed [12]. In brief, both the SPR and GMR optical biosensors are suffering from some essential drawbacks.

Fortunately, the bound-states-in-the-continuum (BICs) optical biosensors have emerged in recent years and can solve these problems. BICs are non-radiative modes coexisting with the continuous spectrum of radiative waves, and theoretically have infinite lifetimes and *Q* values. Importantly, periodic nanostructures inspired high-*Q* BICs modes, which could combine the advantages and compensate the disadvantages of SPR and GMR, support the label-free optical biosensors with higher sensing performance. In addition, although the high-*Q* ability has already been a highlight of BICs, the low-loss integration of BICs chips cannot be ignored [25]. The exceptional points induced by BICs have already been realized in photonic integrated circuits with light confined and guided in the waveguides, which can also benefit biosensing ability [26]. Hence, the BICs biosensing chips featuring high *Q* and easy integration show great potential in a variety of applications, such as biological science, clinical medicine, and environmental monitoring.

Overall, BICs have been detailed in many reviews [27,28,29,30,31,32]. In 2016, Hsu et al. [27] reviewed the basic mechanism of BICs across different materials and different optical waves. In 2019, Koshelev et al. [28] introduced the progress of BICs on single nanostructures and symmetry-broken all-dielectric metasurfaces and their specific application in nonlinear nanophotonics. In 2020, Koshelev et al. [29] explained the mechanisms of BICs and quasi-BICs in different nanostructures and provided an outlook for their applications in high-*Q* devices, device miniaturization, nonlinear enhancement, etc. From 2020 to 2021, Azzam et al. [30] and Joseph et al. [31] also introduced the physical mechanism and device structures of BICs. They gave a comprehensive review of diverse BICs applications, including beam steering, nanocavities, chiral enhancement, directional emission, lasers, guiding and on-chip communication, switches, nonlinear harmonic generation, imaging, field enhancement, photodetection, biosensing, etc. In addition, Chai et al. [32] reviewed three mechanisms and some applications of BICs in 2021. All of these reviews have provided guidelines and ideas for the future study of BICs, as well as the innovative design of BIC devices in various fields.

However, these reviews are mainly focused on a broad introduction to BICs. A comprehensive review on label-free BICs optical biosensors is still absent. Therefore, we aim to review the latest progress in relation to label-free BICs optical biosensors and systematically summarize the existing related works. In this review, we first introduce the history and physical mechanisms of BICs and quasi-BICs. Secondly, we introduce the basic performance characteristics of label-free BICs optical biosensors, including the bulk refractive index (RI) sensitivity, the surface RI sensitivity, the *FOM*, and the detection limit (*DL*). Thirdly, we systematically list the current studies on label-free BICs optical biosensors according to the materials and the structures. Finally, we summarize and discuss the status and challenges of label-free BICs optical biosensors and provide an outlook for their future development.

## 2. Mechanism of BICs and Quasi-BICs

BICs are non-radiative modes in various physical systems that theoretically have infinite lifetimes and *Q* values. As early as 1929, Von Neumann and Wigner first discovered the existence of BICs in electronic systems [33]. However, such electrical BICs have not been experimentally supported. In 2008, Marinica et al. [34] introduced BICs to coupled optical waveguide arrays, opening up the exploration of optical BICs. A few years later, Plotnik et al. [35] added two additional waveguides above and below the optical waveguide array to break the symmetry of the system. This operation enables the decoupling of the bound states from the continuous radiation region, causing the radiation to leak into the additionally added waveguide. These types of bound states associated with structural symmetry are called symmetry-protected BICs (SP-BICs) [35,36,37].

Another type of BIC is the Friedrich-Wintgen BIC (FW-BIC) [4,38,39,40] (or accidental BIC), which was proposed by Friedrich and Wintgen in 1985 [38]. System parameters were modulated to make two resonant channels strongly coupled, leading to the disappearance of the resonance linewidth and the formation of the localized bound states (FW-BICs). In 2013, based on the Friedrich-Wintgen method, Hsu et al. [39] observed FW-BICs in a photonic crystal slab (PhCS) by modulating the angle of the incident light; they experimentally confirmed the existence of special points where the radiation vanished and named these points “embedded eigenvalues”. In addition, there is a third type of BIC depending on the mechanism of suppressing radiation leakage. Such BICs are formed when two identical resonances coupled to a single radiation channel and are called Fabry-Perot BICs [34,41,42]. Ideal BICs exist in lossless systems, so most studies on BICs have been conducted in low-loss dielectric nanostructures. However, BICs on lossy systems have not been ignored. In 2018, a FW-BIC at the “avoided crossing” point of two resonances in a metal-dielectric hybrid system was found by Azzam et al. [40], further confirming the existence of BICs in a lossy system.

**Figure 1:**
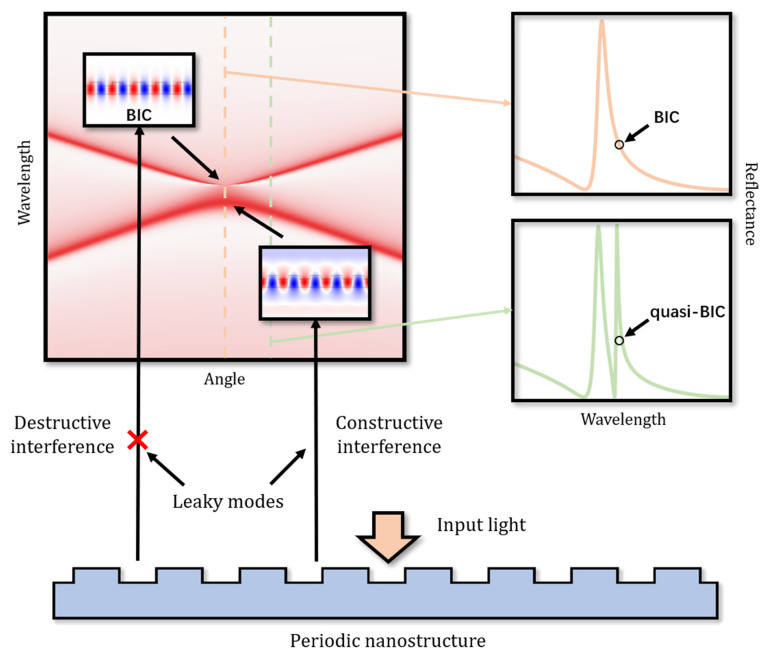
Physical mechanism of BICs and quasi-BICs in a periodic nanostructure.

Figure 1 shows the mechanism of BICs and quasi-BICs. In periodic nanostructures, the band folding of the structures allows the guided modes to leak above the light line, resulting in leaky modes. Ideally, destructive interference between the leaky modes and the radiation modes will lead to BICs. According to this mechanism, BICs theoretically have infinite *Q* values and exist as dark states in the spectrum; this is different from SPRs and GMRs, which are both bright states. However, due to inevitable defects in practice, BICs are no longer ideal bound states but will be transformed into a radiation-leakage state called quasi-BICs with finite *Q* values, also known as supercavity modes [43,44,45,46]. The *Q* values of quasi-BICs are limited by material absorption, structure parameters, fabrication defects, energy leakage, etc. Conversely, these limitations can be utilized to control the *Q* value of quasi-BICs. The most common tunable limitation is the symmetry defect [44]. Moreover, it must be mentioned that quasi-BICs modes also exist in lossy systems, and the influence of materials in such systems cannot be ignored. Such studies are introduced in Section 4.2 and Section 4.3.

**Figure 2:**
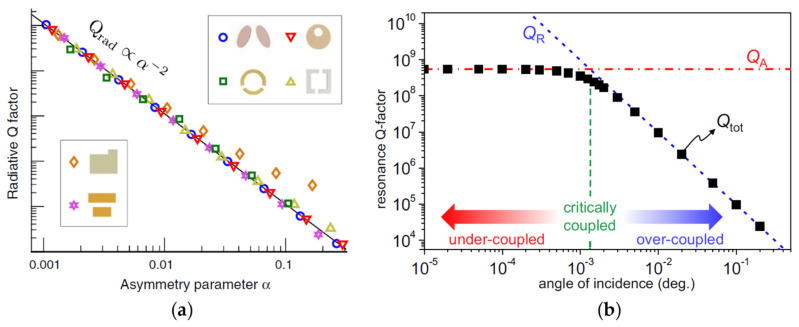
Characteristics of *Q* values of quasi-BICs. (**a**) The relationship between the radiation factor $Q_{R}$ and the asymmetry parameter α in symmetry-broken metasurfaces (reprinted with permission from [44], ©2018, APS); (**b**) the relationships between $Q_{\mathrm{tot}}$, $Q_{R}$, and $Q_{A}$. When $Q_{R}$ tends to infinity, $Q_{\mathrm{tot}}$ is still limited by $Q_{A}$ (reprinted with permission from [36], ©2015, Jae Woong Yoon et al., CC BY license).

Recently, researchers found that the *Q* values of quasi-BICs strongly depend on structure parameters [43,44]. Rybin et al. [43] discussed the supercavity modes in a single silicon nanoresonator and found that the *Q* value of these modes exhibited a power law with the dielectric constant *ε* of the material; this *Q* value was sufficient to produce a strong nonlinear effect at the nanoscale. Koshelev et al. [44] summarized and calculated the *Q* value of various dielectric metasurfaces that were broken with regard to the in-plane inversion symmetry. The asymmetric parameter *α* was set to symbolize the degree of structure asymmetry and the relationship between the radiation component of *Q* ($Q_{\mathrm{rad}}$) and *α* could be described as $Q_{\mathrm{rad}}{=Q}_{0}\alpha^{-2}$, as shown in Figure 2a, where $Q_{0}$ is a constant related to the structure. In addition, the influence of material loss on the *Q* value was considered by Yoon et al. [36]. *Q* values in the silicon grating can be expressed as [36]:

(1) $$\frac{1}{Q_{\mathrm{tot}}}=\frac{1}{Q_{R}}+\frac{1}{Q_{A}},$$

where $Q_{\mathrm{tot}}$ is the total quality factor,$Q_{R}$ is the radiation factor, and $Q_{A}$ is the dissipation factor. Yoon et al. found that $Q_{\mathrm{tot}}$ will reach saturation at $Q_{A}$ when the incident light tends to normal, as shown in Figure 2b. The radiation leakage is completely suppressed, which actually indicates that $Q_{R}$ is divergent, while $Q_{A}$ is still a finite valve due to the unavoidable loss of materials. Briefly, quasi-BICs have the ability to control *Q* values and this ability is quite necessary for high-resolution biosensor designs. Thus, quasi-BICs are expected to support high-*Q*, high-sensitivity, and low-*DL* biodetection through the reasonable setting of structure parameters.

## 3. Performance Characteristics of Label-Free BICs Optical Biosensors

The performance characteristics of optical biosensors are related to their ability to detect analytes in the surrounding medium. For different types of label-free biosensors, unified performance characteristics can be used for evaluation. In this section, a systematic summary of these characteristics is provided.

### 3.1. Bulk RI Sensitivity

Refractive index (RI) change is the basis of biodetection and is closely related to the mechanical, electrical, and optical properties of biology. The bulk RI sensitivity, which reflects the ability of biosensors to detect RI changes in the surrounding medium, is the most widely used performance characteristic of optical biosensors. It can be defined as [1]:

(2) $$S_{\mathrm{bulk}}=\frac{\Delta \lambda }{\Delta n},\;$$

where Δ*n* means the RI change, *λ* is the center wavelength of the optical resonance, and ∆*λ* is the shift of the center wavelength with the RI change. In general, the bulk RI sensitivity of different biosensors varies greatly depending on the materials, the type of optical mode, and the length of the evanescent tail in the surrounding medium.

### 3.2. Surface RI Sensitivity

Although the bulk RI sensitivity can be easily measured by changing the RI of the surrounding medium, it is not the most relevant parameter of a surface-affinity biosensor [12]. Surface RI sensitivity describes the response of the biosensor to the RI change of a thin layer on the surface, which is more representative of the change caused by the binding of proteins or other substances attached to the surface of biosensors. The definition of surface sensitivity is similar to that of bulk sensitivity [12]:

(3) $$S_{\mathrm{surface}}=\frac{\Delta \lambda }{{\Delta n}_{\mathrm{surface}}},\;$$

where ${\Delta n}_{\mathrm{surface}}$ is the RI change of a thin layer on the structure surface. The surface sensitivity of the dielectric nanopore array metasurface proposed by Conteduca et al. [12] is 20 nm/RIU, which is very close to a plasmonic biosensor with the same structure (30 nm/RIU). Even though the bulk sensitivity of the plasmonic structure is much higher than that of the dielectric structure, the surface sensitivity can be very similar. Moreover, some studies have defined the surface sensitivity as the ratio of the central wavelength shift to the thickness of the surface adsorbate layer [47,48].

### 3.3. Figure of Merit

The figure of merit (*FOM*), which combines the sensitivity of the biosensors with the *FWHM*, is the most widely used characteristic of the spectral resolution of optical biosensors. The *FOM* is commonly defined as [21,49]:

(4) $$FOM=\frac{S}{FWHM}.\;$$

However, the *FOM* tends to infinity for biosensors with an infinite *Q* value according to the definition of *Q* and Formula (4), which means the role of sensitivity on the *FOM* can almost be ignored. Expediently, the *FOM* is defined as the product of sensitivity and the *Q* value in some high-*Q* studies [20], which can also fully reflect the sensing ability.

### 3.4. Detection Limit

The detection limit (*DL*) is used to describe the biological resolution of the biosensor. For RI sensing, the *DL* represents the minimum RI change that can be accurately measured, while for biosensing, the *DL* describes the lowest analyte concentration that can be accurately and quantitatively detected by the biosensors. The *DL* is generally defined as the ratio of the sensor resolution (*R*) and sensitivity (*S*) [50]:

(5) $$DL=\frac{R}{S},\;$$

where *R* means the minimum resolution wavelength ($∆\lambda_{\min}\;$ [51]) of the biosensor. The *DL* can also be defined as Formula (6) with the expression of the limit of detection (*LOD*) [1,12]:

(6) $$LOD=\frac{3\sigma }{S_{c}},\;$$

where *σ* is the background noise obtained by measuring the blank sample, and $S_{c}$ is the ratio of the change in the output signal to the change in the corresponding surrounding medium.

The *DL*/*LOD* depends on the sensitivity of the structure to the molecular mass surface coverage, the mass transfer rate to the sensor surface, and the dynamic parameters of the interaction between the analyte and the biometric element. Whether in the plasmonic biosensor [1] or in the dielectric biosensor [12,50], the *DL*/*LOD* values of different types of biosensors are quite different. For high-*Q* structures, the resolution *R* will be very small, which means a smaller *DL*/*LOD*. Therefore, the high-*Q* ability of BICs can contribute to a better sensing performance, allowing biosensors to detect small molecular weight or very low concentration biomolecules. This is a reason why BICs are highly valued in biosensing.

## 4. Label-Free BICs Optical Biosensors Based on Different Materials and Structures

Label-free BICs optical biosensors are expected to be utilized in optical chips with the advantages of both high *Q* and high throughput. Before BICs were widely applied for biosensing, extremely narrow resonances that could be excited by symmetry-broken nanostructures were studied. In 2012, Cetin et al. [15] designed a symmetry-broken golden ring/disk plasmonic metasurface, which could excite a Fano resonance formed by the coupling of an electric quadrupole dark mode and a dipole bright mode. The Fano resonance had excellent sensing performance and was used to detect signal changes caused by the specific binding of the recombinant fusion protein A/G and antibody IgG. In 2013, a symmetry-broken all-dielectric PhCS proposed by Nicolaou et al. [52] was utilized to excite dark states with radiation leakage. The obtained radiation-leaking mode had a high *Q*-value of 10,600 and a sensitivity of over 800 nm/RIU; the high-*Q* value enabled a lower *DL*. In 2014, Yang et al. [53] designed a silicon-based metasurface which enabled coupling between the dark and bright modes; this coupling led to an extremely narrow resonance based on the electromagnetically induced transparency (EIT) effect, which is now believed to have a strong connection with BICs [54,55].

Different from symmetry-breaking studies, the PhCS designed by Liu et al. [56] excited a high-*Q* quasi-BIC resonance by setting the incident angle close to 0°. Both TE and TM polarized light were able to excite the resonances, with the *Q* value tending to infinity at normal incidence. A high *Q*-value of 1.8 × 10^4^ of the TM mode in water was experimentally measured, which showed an excellent biosensing potential.

These early studies unintentionally utilized the concept of BICs. After BIC effect became a research hotspot in respect to biosensors, increasing numbers of studies have emerged. In this section, the latest studies on BICs biosensors in recent years are systematically summarized in terms of the constituent materials and structures.

### 4.1. All-Dielectric BICs

All-dielectric structures that support BICs have been widely used in the design of high-*Q* biosensors based on their advantages of low loss and high functionality. In this section, some all-dielectric BICs biosensors are introduced, including BICs gratings [57,58,59,60,61,62,63], symmetry-broken [64,65,66,67,68,69,70,71,72,73,74,75,76,77] and topological-boundary-states-governed [78] BICs metasurfaces, and BICs PhCSs [56,79,80,81,82,83,84,85,86].

**Figure 3:**
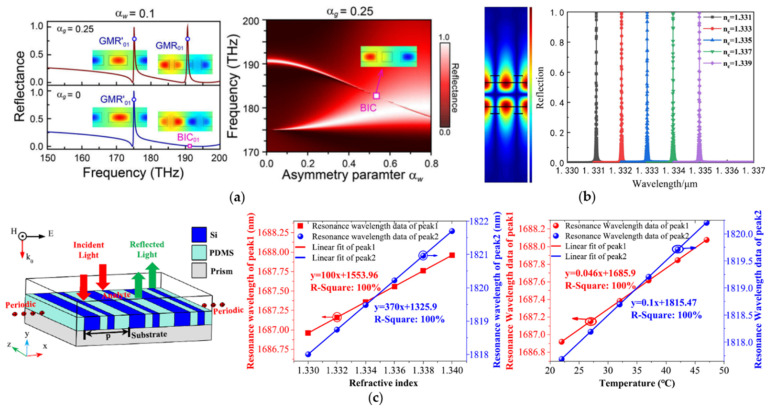
BICs biosensors composed of all-dielectric gratings. (**a**) GMR and BIC modes excited by an asymmetric silicon grating and their Hy field distribution with different degrees of symmetry breaking (reprinted with permission from [57], ©2021, The Optical Society). (**b**) double compound symmetric grating with a localized electric field (reprinted with permission from [58], ©2022, Chinese Optics Letters). (**c**) Si-PDMS symmetry-broken grating with temperature self-compensation function (reprinted with permission from [59], ©2022, IOP).

Grating has become a basic structure of biosensors with its ability to diffract light. Figure 3a shows a symmetry-broken dual silicon grating proposed by Liu et al. [57]. The symmetry degree of the grating is changed by tuning the width and gap of two adjacent grating ridges, and the resonance width is tuned at the same time. Essentially, this setting can make the RI distribution of the grating layer non-uniform within a grating period, so a quasi-BIC with a high FOM can be obtained under specific asymmetric parameters. Based on the BICs-supported dual-grating metamembranes proposed by Hemmati et al. [87], a double compound symmetric grating was designed by Shi et al. [58], as shown in Figure 3b. The resonant linewidth of quasi-BICs was tuned by changing the gap of the grating ridges, and a high FOM of 31,467 can be theoretically achieved. Figure 3c shows a symmetry-broken Si-PDMS grating with the temperature self-compensation function proposed by Wang et al. [59]. Two extremely narrow quasi-BIC resonances can be excited by the grating. One quasi-BIC resonance is sensitive to the RI change, while the other is more sensitive to temperature changes according to the thermo-optic coefficient of the material. The temperature anti-interference ability and the accuracy of the RI sensing can be improved due to these properties.

In addition to the works based on structural asymmetry, a theoretical study of BICs on defect-free periodic gratings was proposed by Maksimov and Dmitrii et al. [60,61]. By using Zel’dovich perturbation theory, they deduced that the theoretical maximum sensitivity of the BIC mode excited by the dielectric grating should be the ratio of the central wavelength of the BIC resonance to the RI of the covering medium; this ratio was independent of the material and the geometric parameters of the grating. A theoretical basis for designing high-performance BICs optical biosensors was provided.

**Figure 4:**
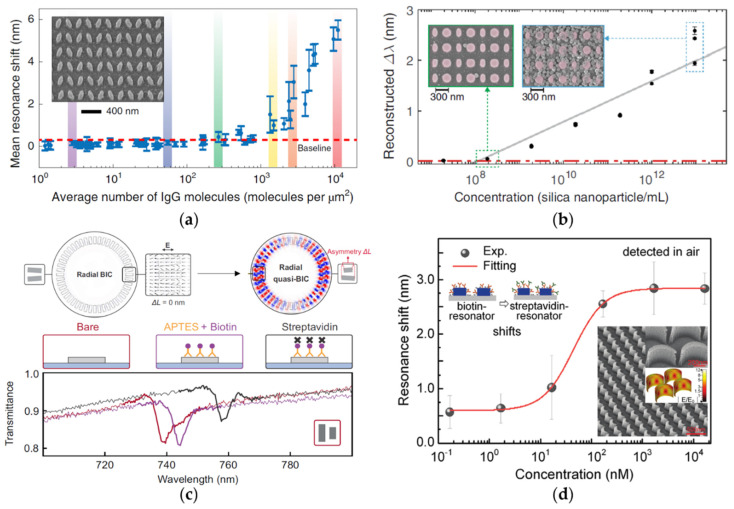
BIC biosensors composed of all-dielectric metasurfaces. (**a**) A symmetry-broken elliptical silicon metasurface for hyperspectral imaging biodetection of IgG (reprinted with permission from [64], ©2019, Springer Nature); (**b**) the reconstructed spectral shift calibration curve of diatomic metasurfaces for detection of biotinylated silica nanoparticles (reprinted with permission from [65], ©2021, Yasaman Jahani et al., CC BY license); (**c**) polarization-independent symmetry-broken ring metasurface for biotin-streptavidin specific binding experiment (reprinted with permission from [66], ©2022, Lucca Kühner et al., CC BY license); (**d**) all-dielectric crescent metasurface for biotin-streptavidin specific binding experiment (reprinted with permission from [67], ©2021, Juan Wang et al., CC BY license).

Metasurfaces are periodic arrays of subwavelength units that can modulate light through scattering [88]. One of the advantages of metasurfaces is their flexibility in structural design, so that they can provide more possibilities for biosensors [89]. Figure 4a shows a symmetry-broken metasurface composed of pairs of tilted silicon nanobars designed by Yesilkoy et al. [64]. In this work, imaging technology was used to record the spectral information of quasi-BICs at different wavelengths. Once the information was decoded, a two-dimensional spatial spectrum in a large area could be obtained, and the imaging points of extremely low-concentration analytes could be observed. All of these studies make great contributions to the biodetection of low-concentration biomolecular signals that are easily covered by background noise. Figure 4b shows another imaging biosensors proposed by Jahani et al. [65]. With similar methods as Yesilkoy et al. [64], a diatomic metasurface was applied to the real-time detection of breast cancer extracellular vesicles (EVs) encompassing exosomes, realizing a real-time biodetection of EVs binding with a low *DL* of 133 femtomolar solutions. A measurement of nanoparticles was also made with detection of on average 0.41 nanoparticles per µm^2^. Figure 4c shows a symmetry-broken toroidal metasurface composed of double rod unit cells designed by Kühner et al. [66]. Structures with an infinite period are generally set to study ideal BICs in simulation, but the *Q* values are commonly limited by the structure size in practice and are not consistent with the simulation *Q*. Fortunately, the toroidal metasurface can ignore the boundary limitation and light polarization, and have been proved to achieve a large resonance shift in the nano-level in biotin-streptavidin specific biodetection. Figure 4d shows an all-dielectric crescent metasurface designed by Wang et al. [67], which has been used for the biodetection of a biotin-streptavidin specific binding. The resonance of quasi-BICs moved 1.5 nm per nanomole (nM) of streptavidin, and the *DL* was as low as 0.167 nM. Meanwhile, a comparison between the sensing performance of the second-order mode and the quasi-BIC basic mode was also made; the sensitivity of the second-order mode could reach twice that of the quasi-BIC basic mode, and also had a narrower linewidth. This property shows a great potential of high-order BICs in biosensing.

**Figure 5:**
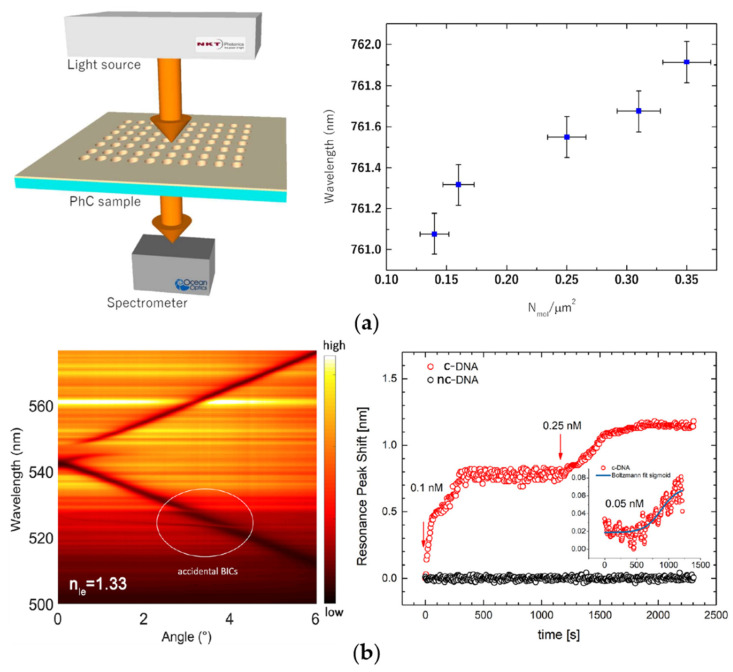
BIC biosensors composed of all-dielectric PhCSs. (**a**) A Si_3_N_4_ PhCS utilized to recognize the interaction between tumor suppressor protein p53 and cancer protein MDM2 (reprinted with permission from [80], ©2018, Silvia Romano et al., CC BY license); (**b**) accidental BICs in PhCS for DNA detection (reprinted with permission from [83], ©2021, Gianluigi Zito et al., CC BY license).

PhCSs have great potential in miniaturization, integration and biodetection combined with microfluidic chips [90]. Romano et al. [80,81,82,91] have been committed to studying the biosensing performance of PhCSs. Figure 5a shows the study of Romano et al. [80] on using resonance-trapped BICs excited by Si_3_N_4_ PhCSs to recognize the interaction between tumor suppressor protein p53 and cancer protein MDM2; this study has great significance for low-cost research with respect to anticancer pharmacological active molecules. Romano et al. [81] experimentally verified the excellent biosensing performance of BICs excited by Si_3_N_4_ PhCSs. Combined with microfluidic technology, the PhCS biosensors with high surface sensitivity could be utilized for the stable biodetection of ultra-low molecular weight particles. Romano et al. [82] then studied the evanescent field sensing mechanism provided by BIC modes in PhCSs. They noticed that the smaller the RI difference between the material of the PhCS and the substrate, the greater the sensitivity of the structure. The PhCS theoretically had the potential to achieve an ultra-high sensitivity (~4000 nm/RIU) at a certain RI distribution. In addition, Romano et al. [91] combined surface fluorescence enhancement with label-free biosensing. Two quasi-BIC modes excited by Si_3_N_4_ PhCSs are used respectively for the laser input amplification and the RI probe to achieve 2D RI imaging for PC3 cells. Furthermore, Figure 5b shows the Si_3_N_4_ PhCS proposed by Zito et al. [83] for DNA biodetection. A FW-BIC can be excited when the light source is incident at a small angle, and the FW-BIC could be utilized for the real-time specific biodetection of DNA.

PhCSs based on silicon material were also proven to have excellent sensing performance. For example, a quasi-BIC with *Q* > 10^7^ in infrared spectroscopy can be theoretically achieved by a coupled double-layer silicon PhCS proposed by Liu et al. [84]. A high *Q*-value of 1.2 × 10^4^ and a low-*DL* of 6 × 10^−5^ was also realized experimentally. In addition, Lv et al. [85] pinpointed the BIC property of a silicon PhCS from the perspective of the topological charge. BICs are essentially topological defects in the momentum space, namely nontrivial topological charge. By tuning the lattice periodicity of the PhCS, the topological charges can be merged, and “merging BICs” can be achieved. Merging BICs are not only sensitive to environmental disturbance, but they are also robust, allowing biosensors to maintain a stable high *Q*-value in different RI environments. A sensitivity 12 times greater than the common isolated BICs can be finally realized.

### 4.2. Metallic BICs

Metal-based SPRs have been widely applied in biosensors due to their excellent RI sensitivity [92]. However, metal has inevitable absorption loss, which will greatly limit its *Q* value. Applying BICs to metallic structures is believed to overcome this difficulty and increase the *Q* and *FOM* values of the metal-based biosensors while ensuring high sensitivity. In this section, we summarize the metallic BICs optical biosensors, including symmetry-broken metallic terahertz (THz) metasurfaces [93,94,95,96,97,98] and symmetry-broken metallic nano-metasurfaces [99].

**Figure 6:**
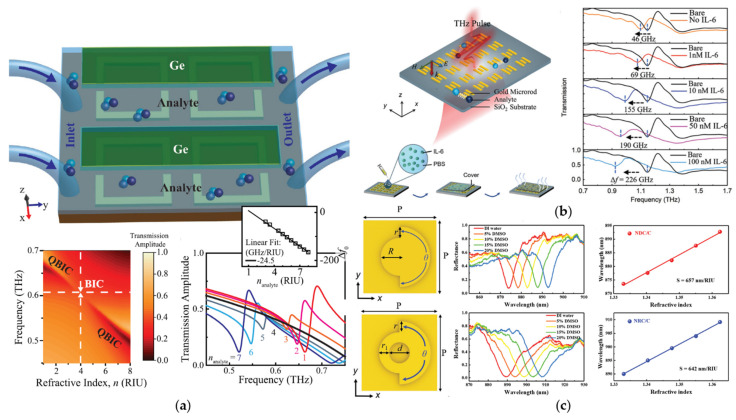
Metallic BIC biosensors. (**a**) A symmetry-broken golden THz metasurface coated with germanium strip and its bulk RI sensing ability (reprinted with permission from [94], ©2021, Wiley-VCH GmbH); (**b**) an ultra-sensitive THz metasurface utilized for biodetection of IL-6 (reprinted with permission from [95], ©2021, RSC); (**c**) a golden nano-metasurface with C-shaped edge and its bulk RI sensing ability (reprinted with permission from [99], ©2021, Gianluigi Zito et al., CC BY license).

BICs-based metallic metasurfaces can achieve good sensing performance in the THz band. Srivastava et al. [93] designed a symmetry-broken golden split ring BIC terahertz metasurface with the cycloolefin copolymer (COC) substrate. This structure achieved a good amplitude sensitivity and could be used for health monitoring and very low-*DL* biodetection. Based on the work of Srivastava et al., a THz metasurface coated with germanium strips of different widths and thicknesses was proposed by Tan et al. [94], as shown in Figure 6a. The germanium strips were utilized to excite quasi-BIC modes, which made the metasurface extremely sensitive and able to realize the functions of narrowband THz optical filters, modulators, and sensors. Figure 6b shows a quasi-BICs ultra-sensitive THz metasurface with three golden bars as the periodic units designed by Wang et al. [95]. A sensitivity of 165 GHz/RIU was achieved. This THz metasurface can be used to detect extremely dilute interleukin-6 (IL-6) solution with a concentration as low as nM.

Metallic BIC metasurfaces also show extraordinary sensing potential at the nanoscale. Figure 6c shows a golden nano-disk/ring metasurface with C-shaped edges designed by Zhou et al. [99]. By modulating the width and the opening angle of the C-shaped edge of the structural units, the dark modes supported by the electric monopole or the electric quadrupole can be excited. The quasi-BICs supported by the electric quadrupole mode have excellent field enhancement ability and bulk sensing performance, and can obviously achieve higher *Q* and *FOM* values than general metallic biosensors. Such a nanoscale all-metal design provides a promising platform for high-sensitivity optical biosensing.

### 4.3. Hybrid Metal-Dielectric BICs

Hybrid metal-dielectric nanostructures have been shown to improve the sensitivity of biosensors with less loss of resolution [100]. In 2018, a hybrid BIC in the coupling structure of a silver grating and dielectric waveguide was observed by Azzam et al. [40]. Essentially, the hybrid BIC is the result of the strong coupling of two (or several) optical resonances (generally resonances of two different mechanisms, i.e., coupling between a SPR and a GMR). Considering their advantages, hybrid structures are included in the design of BICs biosensors. In this section, some hybrid BICs biosensors are summarized, including hybrid metal-dielectric gratings [101,102,103] and metasurfaces [104,105,106].

**Figure 7:**
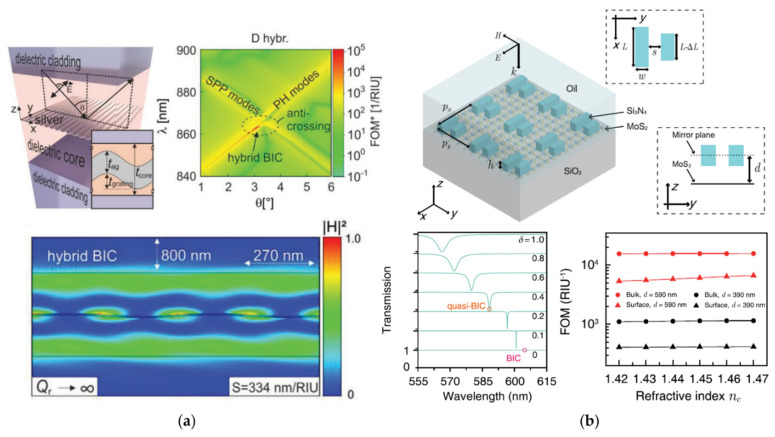
Hybrid metal-dielectric BIC biosensors. (**a**) A sine-modulated silver grating coupled with the dielectric material OrmoCore. Hybrid-BIC occurs at the “avoiding cross” of SPP modes and photonic (PH) modes, and has infinite radiation quality *Q*_r_ (reprinted with permission from [101], ©2020, Wiley-VCH GmbH); (**b**) a symmetry-broken BICs metasurface composed of Si_3_N_4_ symmetry-broken metasurface and MoS_2_ monolayer with excellent sensing ability of maintaining high *FOM* under different RIs (reprinted with permission from [104], © 2022, IOP).

Figure 7a shows the work of Meudt et al. [101]. A sinusoidal modulated silver grating was coupled into a dielectric material, and a hybrid-BIC with a *Q* factor of 2520 near the “avoiding cross” was achieved. The sensitivity of this hybrid BIC is a compromise between the hybrid SPR mode and the hybrid GMR mode, and the *Q* value is much higher than both of them. In addition, the studies of Joseph et al. [107,108] on hybrid gratings need to be mentioned. In 2020, a hybrid structure that involved coating a layer of gold film on the composite photoresist (NPR) grating was proposed by Joseph et al. [107]. However, only hybrid SPP and hybrid GMR modes were discussed, and not hybrid BICs. The *Q* and *FOM* values of these hybrid modes are all in the order of 10. In 2021, a hybrid structure of As_2_Se_3_ sinusoidal grating coupled with gold film was proposed by Joseph et al. [108] and a further discussion on the hybrid BICs was given. The antisymmetric field distribution of the hybrid BIC was observed, and the ability to increase the *Q* value was found; the *Q* value was improved compared to the previous work [107]. Such an ability to enhance the *Q* and *FOM* is necessary for biosensors.

Hybrid metasurfaces are also followed with interests. Figure 7b shows a symmetry-broken metasurface composed of Si_3_N_4_ and MoS_2_ proposed by Wang et al. [104]. Quasi-BICs were used to achieve maximum absorption through adjusting the asymmetric parameter of the units. The metasurface was further used for the bulk RI sensing and an ultra-high *FOM* of more than 10^4^ was theoretically obtained. In addition, some hybrid-BICs studies on achieving ultra-sensitive biosensors in the THz band were also summarized, such as the Al-Si arrow-shaped THz metasurface designed by Liu et al. [105] and the aluminum silicon rectangular hole array THz metasurface designed by Liu et al. [106].

In brief, metallic and hybrid BICs are still at a stage of theoretical simulation and have not been experimentally applied to biodetection; especially, the hybrid BIC is a new solution worth exploring. Evidentially, hybrid BICs have great potential to improve the performance of biosensors and are expected to be a hotspot of BICs biosensors in the future.

## 5. Summary and Outlook

In conclusion, we reviewed the latest studies of label-free BICs optical biosensors in recent years. We introduced label-free BICs optical biosensors in terms of their background, history, and mechanism, and we also compared them with traditional label-free SPR biosensors and GMR biosensors to highlight their advantages. We listed the general performance characteristics of optical biosensors. Finally, we summarized the BICs-biosensing works mentioned in our review for an intuitive comparison, as shown in Table 1.

Label-free BICs optical biosensors are just beginning, bringing new possibilities to break the bottlenecks of the biodetection and other industries. However, although the advantages and contributions of BICs biosensors are undeniable, some challenges cannot be ignored: (1) theoretically, BICs can easily achieve *Q* values exceeding 10^4^, but their *Q* values are still distributed between 10^2^ and 10^4^ experimentally; (2) although *Q* value can be tuned by structure asymmetry parameters, it still depends on the highly symmetric structures to obtain quasi-BICs with extremely high *Q*-values. Generally, a small angle of incident light is required for detecting such high-*Q* quasi-BICs. However, such settings need high system accuracy and environmental stability, which is not conducive to the practicality of products; (3) biosensors are mostly tested in a laboratory, and the detection object is usually a single marker in an ideal environment (i.e., normal saline), while real biological liquid environments (i.e., blood, urine, etc.) are more complex. Meanwhile, different kinds of biomolecules with similar structures also bring a serious challenge to the biochemical surface functionalization technology and the specific capture ability of biosensors; (4) BICs nanostructures with stable high *Q*-values require high accuracy. However, the current manufacturing technology is difficult to achieve precise geometric differences, such as several nanometers. How to balance the structure design and the manufacturing error is also a problem needed to be considered.

Some existing studies have provided solutions. The operation of Lv et al. [85] of merging BICs can achieve higher *Q* values with better robustness and can ensure high *Q*-values above 10^4^ both in air and in solution. Such robustness is particularly important in resisting manufacturing errors and obtaining a more stable biodetection environment. However, the PhCS used for merging BICs proposed by Lv et al. is not sensitive enough (only 36 nm/RIU), so hybrid metal-dielectric structures can be considered to achieve higher sensitivity. At present, hybrid BICs have not been practically applied, but they have gradually attracted attention. Furthermore, quasi-BICs essentially involve optical resonant phenomena, so the phase will also change with the resonant wavelength shifts. Phase-change detection is more sensitive and is expected to have a lower *DL* [11], which is also a biodetection method worth studying.

## Figures and Tables

**Table 1:** Summary of different label-free BICs optical biosensors.

Structure	Analyte	*Q* Factor	Bulk Sensitivity (nm/RIU)	*FOM* (RIU^−1^)	*DL*/*LOD*	Ref.
All-Dielectric Gratings			345	2622		[49]
		441	1506	~5000		[57]
			472	31,467		[58]
			369.43	3212.43	1.56 × 10^−5^ RIU	[59]
		3 × 10^6^	656	1.64 × 10^6^		[62]
		12,620	31,394	1000		[63]
All-Dielectric Metasurfaces	Oil	483	379	103		[53]
	M-IgG	144	263		<3 molecules/µm^2^	[64]
	Nanoparticles; EVs	178.6	305	68	0.41 molecules/µm^2^; 133 femtomolar	[65]
	Biotin-streptavidin	120	326		0.167 nM	[67]
	Exosomes	750	440	677		[68]
	Biotin-streptavidin	500		20		[66]
	ErbB_2_	900	720		0.7 ng/L	[69]
		17,684	630			[70]
		3.15 × 10^4^	295	738		[71]
			342	1295		[72]
		20,561	170.58 (GHz/RIU)			[73]
			155	387,500		[74]
		14,437	394	4925		[75]
		2617	300	440		[76]
	SOG, PMMA-A4, ZEP520A	~10^2^	608	46		[77]
		1045	100	145		[78]
All-Dielectric PhCSs	Cargille RI liquids	10,643	832		~10^−8^ RIU	[52]
	Ethanol/DI	3.2 × 10^4^	94.5		3 × 10^−5^ RIU	[56]
		10^4^~10^5^	312.8	~10^3^		[79]
	p53-MDM2	760			66 nM	[80]
All-Dielectric PhCSs	BPT/ethanol solution	2000	178	445	186 Da	[81]
	Cargille RI liquids		226	258	4 × 10^−7^ RIU	[82]
	PNA-DNA	~10^2^			0.05 nM	[83]
	Ethanol/DI	1.2 × 10^4^	94		6 × 10^−5^ RIU	[84]
	Cargille RI liquids	>7 × 10^4^	36	5990	~10^−5^ RIU	[85]
		25,643	148	821		[86]
	PC3 cell	905	102.6		~10^−5^ RIU	[91]
Plasmonic Metasurfaces	D/A-IgG		648	72		[15]
	Glycerol/water		282	4		[16]
	Ge film		0.28 (/RIU)			[93]
	Ge film		1.7 × 10^4^			[94]
	IL-6	16	165 (GHz/RIU)		~1 nM	[95]
		1016	775.7 (GHz/RIU)	284		[96]
			200 (GHz/RIU)			[97]
		64	265.06 (GHz/RIU)	9.1		[98]
	DMSO/DI	145	657	109		[99]
Hybrid Gratings	Air	1300	334	1.43 × 10^5^		[101]
		111	~500	100		[102]
	Glucose solution	~5000	1264	7022		[103]
Hybrid Metasurfaces			157	15,570		[104]
		≥250	11.1 (GHz/RIU)			[105]
		1130	9.41 (GHz/RIU)			[106]

## References

[1] Spackova B., Wrobel P., Bockova M., Homola J. (2016). Optical Biosensors Based on Plasmonic Nanostructures: A Review. Proc. IEEE.

[2] Inan H., Poyraz M., Inci F., Lifson M.A., Baday M., Cunningham B.T., Demirci U. (2017). Photonic crystals: Emerging biosensors and their promise for point-of-care applications. Chem. Soc. Rev..

[3] Altug H., Oh S.H., Maier S.A., Homola J. (2022). Advances and applications of nanophotonic biosensors. Nat. Nanotechnol..

[4] Yang Y., Peng C., Liang Y., Li Z.B., Noda S. (2014). Analytical Perspective for Bound States in the Continuum in Photonic Crystal Slabs. Phys. Rev. Lett..

[5] Zhuo Y., Hu H., Chen W.L., Lu M., Tian L.M., Yu H.J., Long K.D., Chow E., King W.P., Singamaneni S. (2014). Single nanoparticle detection using photonic crystal enhanced microscopy. Analyst.

[6] Luan E.X., Shoman H., Ratner D.M., Cheung K.C., Chrostowski L. (2018). Silicon Photonic Biosensors Using Label-Free Detection. Sensors.

[7] Karmakar S., Kumar D., Varshney R.K., Chowdhury D.R. (2020). Strong terahertz matter interaction induced ultrasensitive sensing in Fano cavity based stacked metamaterials. J. Phys. D Appl. Phys..

[8] Xu Y., Bai P., Zhou X.D., Akimov Y., Png C.E., Ang L.K., Knoll W., Wu L. (2019). Optical Refractive Index Sensors with Plasmonic and Photonic Structures: Promising and Inconvenient Truth. Adv. Opt. Mater..

[9] Kazanskiy N.L., Khonina S.N., Butt M.A. (2020). Plasmonic sensors based on Metal-insulator-metal waveguides for refractive index sensing applications: A brief review. Physica E.

[10] Liu J.J., Jalali M., Mahshid S., Wachsmann-Hogiu S. (2020). Are plasmonic optical biosensors ready for use in point-of-need applications?. Analyst.

[11] Pitruzzello G., Krauss T.F. (2018). Photonic crystal resonances for sensing and imaging. J. Opt..

[12] Conteduca D., Barth I., Pitruzzello G., Reardon C.P., Martins E.R., Krauss T.F. (2021). Dielectric nanohole array metasurface for high-resolution near-field sensing and imaging. Nat. Commun..

[13] Triggs G.J., Wang Y., Reardon C.P., Fischer M., Evans G.J.O., Krauss T.F. (2017). Chirped guided-mode resonance biosensor. Optica.

[14] Kenaan A., Li K.Z., Barth I., Johnson S., Song J., Krauss T.F. (2020). Guided mode resonance sensor for the parallel detection of multiple protein biomarkers in human urine with high sensitivity. Biosens. Bioelectron..

[15] Cetin A.E., Altug H. (2012). Fano Resonant Ring/Disk Plasmonic Nanocavities on Conducting Substrates for Advanced Biosensing. ACS Nano.

[16] Zhang Q., Wen X.L., Li G.Y., Ruan Q.F., Wang J.F., Xiong Q.H. (2013). Multiple Magnetic Mode-Based Fano Resonance in Split-Ring Resonator/Disk Nanocavities. ACS Nano.

[17] Cetin A.E., Etezadi D., Galarreta B.C., Busson M.P., Eksioglu Y., Altug H. (2015). Plasmonic Nanohole Arrays on a Robust Hybrid Substrate for Highly Sensitive Label-Free Biosensing. ACS Photonics.

[18] Arora P., Talker E., Mazurski N., Levy U. (2018). Dispersion engineering with plasmonic nano structures for enhanced surface plasmon resonance sensing. Sci. Rep..

[19] Kabashin A.V., Evans P., Pastkovsky S., Hendren W., Wurtz G.A., Atkinson R., Pollard R., Podolskiy V.A., Zayats A.V. (2009). Plasmonic nanorod metamaterials for biosensing. Nat. Mater..

[20] Ge C., Lu M., George S., Flood T.A., Wagner C., Zheng J., Pokhriyal A., Eden J.G., Hergenrother P.J., Cunningham B.T. (2013). External cavity laser biosensor. Lab Chip.

[21] Zhou Y., Wang B.W., Guo Z.H., Wu X. (2019). Guided Mode Resonance Sensors with Optimized Figure of Merit. Nanomaterials.

[22] Ciminelli C., Campanella C.M., Dell’Olio F., Campanella C.E., Armenise M.N. (2013). Label-free optical resonant sensors for biochemical applications. Prog. Quantum Electron..

[23] Zhou X.Y., Zhang L., Armani A.M., Zhang D.H., Duan X.X., Liu J., Zhang H., Pang W. (2014). On-Chip Biological and Chemical Sensing With Reversed Fano Lineshape Enabled by Embedded Microring Resonators. IEEE J. Sel. Top. Quantum.

[24] Liao J., Wu X., Liu L.Y., Xu L. (2016). Fano resonance and improved sensing performance in a spectral-simplified optofluidic micro-bubble resonator by introducing selective modal losses. Opt. Express.

[25] Yu Z.J., Xi X., Ma J.W., Tsang H.K., Zou C.L., Sun X.K. (2019). Photonic integrated circuits with bound states in the continuum. Optica.

[26] Qin H.Y., Shi X.D., Ou H.Y. (2022). Exceptional points at bound states in the continuum in photonic integrated circuits. Nanophotonics.

[27] Hsu C.W., Zhen B., Stone A.D., Joannopoulos J.D., Soljacic M. (2016). Bound states in the continuum. Nat. Rev. Mater..

[28] Koshelev K., Bogdanov A., Kivshar Y. (2019). Meta-optics and bound states in the continuum. Sci. Bull..

[29] Koshelev K., Bogdanov A., Kivshar Y. (2020). Engineering with Bound States in the Continuum. Opt. Photonics News.

[30] Azzam S.I., Kildishev A.V. (2021). Photonic Bound States in the Continuum: From Basics to Applications. Adv. Opt. Mater..

[31] Joseph S., Pandey S., Sarkar S., Joseph J. (2021). Bound states in the continuum in resonant nanostructures: An overview of engineered materials for tailored applications. Nanophotonics.

[32] Chai R.H., Liu W.W., Cheng H., Tian J.G., Chen S.Q. (2021). Bound States of Continuumin Optical Artificial Micro-Nanostructures: Fundamentals Developments and Applications. Acta Opt. Sin..

[33] von Neumann J., Wigner E. (1929). Concerning the behaviour of eigenvalues in adiabatic processes. Physica Z.

[34] Marinica D.C., Borisov A.G., Shabanov S.V. (2008). Bound states in the continuum in photonics. Phys. Rev. Lett..

[35] Plotnik Y., Peleg O., Dreisow F., Heinrich M., Nolte S., Szameit A., Segev M. (2011). Experimental Observation of Optical Bound States in the Continuum. Phys. Rev. Lett..

[36] Yoon J.W., Song S.H., Magnusson R. (2015). Critical field enhancement of asymptotic optical bound states in the continuum. Sci. Rep..

[37] Cong L.Q., Singh R. (2019). Symmetry-Protected Dual Bound States in the Continuum in Metamaterials. Adv. Opt. Mater..

[38] Friedrich H., Wintgen D. (1985). Interfering Resonances and Bound-States in the Continuum. Phys. Rev. A.

[39] Hsu C.W., Zhen B., Lee J., Chua S.L., Johnson S.G., Joannopoulos J.D., Soljacic M. (2013). Observation of trapped light within the radiation continuum. Nature.

[40] Azzam S.I., Shalaev V.M., Boltasseva A., Kildishev A.V. (2018). Formation of Bound States in the Continuum in Hybrid Plasmonic-Photonic Systems. Phys. Rev. Lett..

[41] Longhi S. (2007). Bound states in the continuum in a single-level Fano-Anderson model. Eur. Phys. J. B.

[42] Weimann S., Xu Y., Keil R., Miroshnichenko A.E., Tunnermann A., Nolte S., Sukhorukov A.A., Szameit A., Kivshar Y.S. (2013). Compact Surface Fano States Embedded in the Continuum of Waveguide Arrays. Phys. Rev. Lett..

[43] Rybin M.V., Koshelev K.L., Sadrieva Z.F., Samusev K.B., Bogdanov A.A., Limonov M.F., Kivshar Y.S. (2017). High-Q Supercavity Modes in Subwavelength Dielectric Resonators. Phys. Rev. Lett..

[44] Koshelev K., Lepeshov S., Liu M.K., Bogdanov A., Kivshar Y. (2018). Asymmetric Metasurfaces with High-Q Resonances Governed by Bound States in the Continuum. Phys. Rev. Lett..

[45] Bogdanov A.A., Koshelev K.L., Kapitanova P.V., Rybin M.V., Gladyshev S.A., Sadrieva Z.F., Samusev K.B., Kivshar Y.S., Limonov M.F. (2019). Bound states in the continuum and Fano resonances in the strong mode coupling regime. Adv. Photonics.

[46] Chen W.J., Chen Y.T., Liu W. (2019). Multipolar Conversion Induced Subwavelength High-Q Kerker Supermodes with Unidirectional Radiations. Laser Photonics Rev..

[47] Lu X.Y., Zhang T.Y., Wan R.G., Xu Y.T., Zhao C.H., Guo S. (2018). Numerical investigation of narrowband infrared absorber and sensor based on dielectric-metal metasurface. Opt. Express.

[48] Zhang C.R., Zhou Y., Mi L., Ma J., Wu X., Fei Y.Y. (2021). High Performance of a Metal Layer-Assisted Guided-Mode Resonance Biosensor Modulated by Double-Grating. Biosensors.

[49] Lu H., Huang M., Kang X.B., Liu W.X., Dong C., Zhang J., Xia S.Q., Zhang X.Z. (2018). Improving the sensitivity of compound waveguide grating biosensor via modulated wavevector. Appl. Phys. Express.

[50] White I.M., Fan X.D. (2008). On the performance quantification of resonant refractive index sensors. Opt. Express.

[51] Zhang Y.N., Zhao Y., Lv R.Q. (2015). A review for optical sensors based on photonic crystal cavities. Sens. Actuat A Phys..

[52] Nicolaou C., Lau W.T., Gad R., Akhavan H., Schilling R., Levi O. (2013). Enhanced detection limit by dark mode perturbation in 2D photonic crystal slab refractive index sensors. Opt. Express.

[53] Yang Y.M., Kravchenko I.I., Briggs D.P., Valentine J. (2014). All-dielectric metasurface analogue of electromagnetically induced transparency. Nat. Commun..

[54] He F.Y., Liu J.J., Pan G.M., Shu F.Z., Jing X.F., Hong Z. (2021). Analogue of Electromagnetically Induced Transparency in an All-Dielectric Double-Layer Metasurface Based on Bound States in the Continuum. Nanomaterials.

[55] Algorri J.F., Dell’Olio F., Roldan-Varona P., Rodriguez-Cobo L., Lopez-Higuera J.M., Sanchez-Pena J.M., Dmitriev V., Zografopoulos D.C. (2022). Analogue of electromagnetically induced transparency in square slotted silicon metasurfaces supporting bound states in the continuum. Opt. Express.

[56] Liu Y.H., Wang S.L., Zhao D.Y., Zhou W.D., Sun Y.Z. (2017). High quality factor photonic crystal filter at k approximate to 0 and its application for refractive index sensing. Opt. Express.

[57] Liu C.B., Bai Y., Zhou J., Chen J.H., Qiao L.J. (2021). Refractive index sensing by asymmetric dielectric gratings with both bound states in the continuum and guided mode resonances. Opt. Express.

[58] Shi C.Y., Liu X.H., Hu J.H., Han H.Y., Zhao J.J. (2022). High performance optical sensor based on double compound symmetric gratings. Chin. Opt. Lett..

[59] Wang Q., Jiang J.X., Wang L., Yin X.Y., Yan X., Zhu A.S., Qiu F.M., Zhang K.K. (2022). An asymmetric grating refractive index sensor generating quasi-bound states in the continuum with high figure of merit and temperature self-compensation. J. Phys. D Appl. Phys..

[60] Maksimov D.N., Gerasimov V.S., Romano S., Polyutov S.P. (2020). Refractive index sensing with optical bound states in the continuum. Opt. Express.

[61] Maksimov D.N., Gerasimov V.S., Bogdanov A.A., Polyutov S.P. (2022). Enhanced sensitivity of an all-dielectric refractive index sensor with an optical bound state in the continuum. Phys. Rev. A.

[62] Mesli S., Yala H., Hamidi M., BelKhir A., Baida F.I. (2021). High performance for refractive index sensors via symmetry-protected guided mode resonance. Opt. Express.

[63] Zhao J., Wang J.X., Lai L.P., Su Q.Q., Qiu W.B., Zhuo L.Q. (2022). High-Q Dual-Band Terahertz Sensor Based on All-Dielectric Metasurface. Laser Optoelectron. Prog..

[64] Yesilkoy F., Arvelo E.R., Jahani Y., Liu M.K., Tittl A., Cevher V., Kivshar Y., Altug H. (2019). Ultrasensitive hyperspectral imaging and biodetection enabled by dielectric metasurfaces. Nat. Photonics.

[65] Jahani Y., Arvelo E.R., Yesilkoy F., Koshelev K., Cianciaruso C., De Palma M., Kivshar Y., Altug H. (2021). Imaging-based spectrometer-less optofluidic biosensors based on dielectric metasurfaces for detecting extracellular vesicles. Nat. Commun..

[66] Kuhner L., Sortino L., Berte R., Wang J., Ren H., Maier S.A., Kivshar Y., Tittl A. (2022). Radial bound states in the continuum for polarization-invariant nanophotonics. Nat. Commun..

[67] Wang J., Kuhne J., Karamanos T., Rockstuhl C., Maier S.A., Tittl A. (2021). All-Dielectric Crescent Metasurface Sensor Driven by Bound States in the Continuum. Adv. Funct. Mater..

[68] Ndao A., Hsu L.Y., Cai W., Ha J.H., Park J., Contractor R., Lo Y.W., Kant B. (2020). Differentiating and quantifying exosome secretion from a single cell using quasi-bound states in the continuum. Nanophotonics.

[69] Wang Y., Ali M.A., Chow E.K.C., Dong L., Lu M. (2018). An optofluidic metasurface for lateral flow-through detection of breast cancer biomarker. Biosens. Bioelectron..

[70] Li B., Yao J., Zhu H., Cai G.X., Liu Q.H. (2021). Asymmetric excitations of toroidal dipole resonance and the magnetic dipole quasi-bound state in the continuum in an all-dielectric metasurface. Opt. Mater. Express.

[71] Yang L., Yu S.L., Li H., Zhao T.G. (2021). Multiple Fano resonances excitation on all-dielectric nanohole arrays metasurfaces. Opt. Express.

[72] Huo Y.Y., Zhang X., Yan M., Sun K., Jiang S.Z., Ning T.Y., Zhao L.N. (2022). Highly-sensitive sensor based on toroidal dipole governed by bound state in the continuum in dielectric non-coaxial core-shell cylinder. Opt. Express.

[73] Li Z.F., Xiang Y.J., Xu S.X., Dai X.Y. (2022). Ultrasensitive terahertz sensing in all-dielectric asymmetric metasurfaces based on quasi-BIC. J. Opt. Soc. Am. B.

[74] Samadi M., Abshari F., Algorri J.F., Roldan-Varona P., Rodriguez-Cobo L., Lopez-Higuera J.M., Sanchez-Pena J.M., Zografopoulos D.C., Dell’Olio F. (2022). All-Dielectric Metasurface Based on Complementary Split-Ring Resonators for Refractive Index Sensing. Photonics.

[75] Wang Y.S., Yu S.L., Gao Z.A., Song S.Z., Li H.Y., Zhao T.G., Hu Z.H. (2022). Excitations of Multiple Fano Resonances Based on Permittivity-Asymmetric Dielectric Meta-Surfaces for Nano-Sensors. IEEE Photonics J..

[76] Yu S.L., Li H., Wang Y.S., Gao Z., Zhao T.G., Yu J.G. (2021). Multiple Fano resonance excitation of all-dielectric nanoholes cuboid arrays in near infrared region. Results Phys..

[77] Hsiao H.H., Hsu Y.C., Liu A.Y., Hsieh J.C., Lin Y.H. (2022). Ultrasensitive Refractive Index Sensing Based on the Quasi-Bound States in the Continuum of All-Dielectric Metasurfaces. Adv. Opt. Mater..

[78] Gu Z.D., Chen J.X., Gao B.F., Wu W., Zhao Z.Y., Cai W., Zhang X.Z., Ren M.X., Xu J.J. (2022). Metasurfaces with high-Q resonances governed by topological edge state. Opt. Lett..

[79] Chao M.H., Liu Q.S., Zhang W.J., Zhuang L.Y., Song G.F. (2022). Mutual coupling of corner-localized quasi-BICs in high-order topological PhCs and sensing applications. Opt. Express.

[80] Romano S., Lamberti A., Masullo M., Penzo E., Cabrini S., Rendina I., Mocella V. (2018). Optical Biosensors Based on Photonic Crystals Supporting Bound States in the Continuum. Materials.

[81] Romano S., Zito G., Torino S., Calafiore G., Penzo E., Coppola G., Cabrini S., Rendina I., Mocella V. (2018). Label-free sensing of ultralow-weight molecules with all-dielectric metasurfaces supporting bound states in the continuum. Photonics Res..

[82] Romano S., Zito G., Yepez S.N.L., Cabrini S., Penzo E., Coppola G., Rendina I., Mocella V. (2019). Tuning the exponential sensitivity of a bound-state-in-continuum optical sensor. Opt. Express.

[83] Zito G., Sanita G., Alulema B.G., Yepez S.N.L., Lanzio V., Riminucci F., Cabrini S., Moccia M., Avitabile C., Lamberti A. (2021). Label-free DNA biosensing by topological light confinement. Nanophotonics.

[84] Liu Y.H., Zhou W.D., Sun Y.Z. (2017). Optical Refractive Index Sensing Based on High-Q Bound States in the Continuum in Free-Space Coupled Photonic Crystal Slabs. Sensors.

[85] Lv J.X., Chen Z.H., Yin X.F., Zhang Z.X., Hu W.W., Peng C. (2020). High-Sensitive Refractive Index Sensing Enabled by Topological Charge Evolution. IEEE Photonics J..

[86] Wang Z., Xue Q., Zhao S.L., Zhang X.R., Liu H.M., Sun X.H. (2022). Study on the characteristics of a photonic crystal sensor with rectangular lattice based on bound states in the continuum. J. Phys. D Appl. Phys..

[87] Hemmati H., Magnusson R. (2019). Resonant Dual-Grating Metamembranes Supporting Spectrally Narrow Bound States in the Continuum. Adv. Opt. Mater..

[88] Qin J., Jiang S.B., Wang Z.S., Cheng X.B., Li B.J., Shi Y.Z., Tsai D.P., Liu A.Q., Huang W., Zhu W.M. (2022). Metasurface Micro/Nano-Optical Sensors: Principles and Applications. ACS Nano.

[89] Zhang S.Y., Wong C.L., Zeng S.W., Bi R.Z., Tai K., Dholakia K., Olivo M. (2021). Metasurfaces for biomedical applications: Imaging and sensing from a nanophotonics perspective. Nanophotonics.

[90] Shi Q., Zhao J.L., Liang L.J. (2021). Two dimensional photonic crystal slab biosensors using label free refractometric sensing schemes: A review. Prog. Quantum Electron..

[91] Romano S., Mangini M., Penzo E., Cabrini S., De Luca A.C., Rendina I., Mocella V., Zito G.L.G. (2020). Ultrasensitive Surface Refractive Index Imaging Based on Quasi-Bound States in the Continuum. ACS Nano.

[92] Wang Z.B., Chen J.J., Khan S.A., Li F.J., Shen J.Q., Duan Q.L., Liu X.Y., Zhu J.F. (2022). Plasmonic Metasurfaces for Medical Diagnosis Applications: A Review. Sensors.

[93] Srivastava Y.K., Ako R.T., Gupta M., Bhaskaran M., Sriram S., Singh R. (2019). Terahertz sensing of 7 nm dielectric film with bound states in the continuum metasurfaces. Appl. Phys. Lett..

[94] Tan T.C., Srivastava Y.K., Ako R.T., Wang W.H., Bhaskaran M., Sriram S., Al–Naib I., Plum E., Singh R. (2021). Active Control of Nanodielectric-Induced THz Quasi-BIC in Flexible Metasurfaces: A Platform for Modulation and Sensing. Adv. Mater..

[95] Wang R., Xu L., Wang J.Y., Sun L., Jiao Y.N., Meng Y., Chen S., Chang C., Fan C.H. (2021). Electric Fano resonance-based terahertz metasensors. Nanoscale.

[96] Chen X., Fan W.H., Jiang X.Q., Yan H. (2022). High-Q Toroidal Dipole Metasurfaces Driven By Bound States in the Continuum for Ultrasensitive Terahertz Sensing. J. Lightwave Technol..

[97] Hu Y.L., Xie S.X., Bai C.J., Shen W.W., Yang J.C. (2022). Quasi-Bound States in the Continuum Enabled Strong Terahertz Chiroptical Response in Bilayer Metallic Metasurfaces. Crystals.

[98] Cen W.Y., Lang T.T., Hong Z., Liu J.J., Xiao M.Y., Zhang J.H., Yu Z.Y. (2022). Ultrasensitive Flexible Terahertz Plasmonic Metasurface Sensor Based on Bound States in the Continuum. IEEE Sens. J..

[99] Zhou Y., Guo Z.H., Zhao X.Y., Wang F.L., Yu Z.Y., Chen Y.Z., Liu Z.R., Zhang S.Y., Sun S.L., Wu X. (2022). Dual-Quasi Bound States in the Continuum Enabled Plasmonic Metasurfaces. Adv. Opt. Mater..

[100] Sarkar S., Gupta V., Kumar M., Schubert J., Probst P.T., Joseph J., Konig T.A.F. (2019). Hybridized Guided-Mode Resonances via Colloidal Plasmonic Self-Assembled Grating. ACS Appl. Mater. Interfaces.

[101] Meudt M., Bogiadzi C., Wrobel K., Gorrn P. (2020). Hybrid Photonic-Plasmonic Bound States in Continuum for Enhanced Light Manipulation. Adv. Opt. Mater..

[102] Chen H.R., Wang H.F., Wong K.Y., Lei D.Y. (2022). High-Q localized surface plasmon resonance based on bound states in the continuum for enhanced refractive index sensing. Opt. Lett..

[103] Tang S.L., Chang C., Zhou P.J., Zou Y. (2022). Numerical Study on a Bound State in the Continuum Assisted Plasmonic Refractive Index Sensor. Photonics.

[104] Wang J., Yang J.Z., Zhao H.W., Chen M. (2021). Quasi-BIC-governed light absorption of monolayer transition-metal dichalcogenide-based absorber and its sensing performance. J. Phys. D Appl. Phys..

[105] Liu X.Y., Li F.Y., Li Y.X., Tang T.T., Liao Y.L., Lu Y.C., Wen Q.Y. (2022). Terahertz metasurfaces based on bound states in the continuum (BIC) for high-sensitivity refractive index sensing. Optik.

[106] Liu W.Y., Li W., Liu C.X., Xing E.B., Zhou Y.R., Liu L., Shi Y.B., Tang J. (2022). All-Optical Tuning of Fano Resonance for Quasi-BIC and Terahertz Sensing Applications. Appl. Sci..

[107] Joseph S., Sarkar S., Joseph J. (2020). Grating-Coupled Surface Plasmon-Polariton Sensing at a Flat Metal-Analyte Interface in a Hybrid-Configuration. ACS Appl. Mater. Interfaces.

[108] Joseph S., Sarkar S., Khan S., Joseph J. (2021). Exploring the Optical Bound State in the Continuum in a Dielectric Grating Coupled Plasmonic Hybrid System. Adv. Opt. Mater..

